# Efficacy of concurrent chemoradiotherapy in subgroups of stage III nasopharyngeal carcinoma: an analysis based on 10-year follow-up

**DOI:** 10.1186/s13014-021-01929-9

**Published:** 2021-11-06

**Authors:** Lei Wang, Zheng Wu, Wanqin Cheng, Dehuan Xie, Feifei Lin, Liangping Xia, Yong Su

**Affiliations:** 1 Department of VIP Region, Sun Yat-sen University Cancer Center, State Key Laboratory of Oncology in South China, Collaborative Innovation Center for Cancer Medicine, Guangdong Key Laboratory of Nasopharyngeal Carcinoma Diagnosis and Therapy, No. 651 Dongfeng Road East, Guangzhou, 510060 People’s Republic of China; 2 Department of Radiation Oncology, Hunan Cancer Hospital and The Affiliated Cancer Hospital of Xiangya School of Medicine, Central South University, 283 Tong Zi Po Road, Changsha, 410013 People’s Republic of China; 3 Department of Radiation Oncology, Shunde Hospital of Southern Medical University, Foshan, 528399 People’s Republic of China; 4 Department of Radiation Oncology, Sun Yat-sen University Cancer Center, State Key Laboratory of Oncology in South China, Collaborative Innovation Center for Cancer Medicine, Guangdong Key Laboratory of Nasopharyngeal Carcinoma Diagnosis and Therapy, No. 651 Dongfeng Road East, Guangzhou, 510060 People’s Republic of China; 5 Department of Radiation Nasopharyngeal Carcinoma, Sun Yat-sen University Cancer Center, State Key Laboratory of Oncology in South China, Collaborative Innovation Center for Cancer Medicine, Guangdong Key Laboratory of Nasopharyngeal Carcinoma Diagnosis and Therapy, No. 651 Dongfeng Road East, Guangzhou, 510060 People’s Republic of China

**Keywords:** Stage III, Nasopharyngeal carcinoma, Intensity-modulated radiotherapy, Concurrent chemoradiotherapy, Survival outcome

## Abstract

**Purpose:**

To evaluate the efficacy of concurrent chemoradiotherapy (CCRT) in subgroups of stage III nasopharyngeal carcinoma (NPC) in the context of intensity-modulated radiotherapy (IMRT).

**Methods:**

A total of 272 patients with stage III NPC who underwent IMRT with or without concurrent chemotherapy were retrospectively reviewed. Clinicopathological features were evaluated by a Cox regression model to identify independent prognostic factors. Survival outcomes were assessed using the Kaplan–Meier method and log-rank test.

**Results:**

The median follow-up time was 108 months. The 10-year locoregional-free survival (LRFS), distant metastasis-free survival (DMFS), disease-free survival (DFS), and overall survival (OS) rates were 87.8%, 80.7%, 68.8%, and 74.9%, respectively. Multivariate analysis showed that the N classification was significantly associated with DMFS (hazard ratio [HR] 3.616, 95% confidence interval [CI] 1.387–9.428, *P* = 0.009), DFS (HR 2.417, 95% CI 1.291–4.423, *P* = 0.006), and OS (HR 3.024, 95% CI 1.385–6.602, *P* = 0.005). In patients with T1-3N2 disease, CCRT was associated with improved 10-year LRFS (89.6% vs. 65.4%, *P* = 0.005), DFS (71.9% vs. 39.4% *P* = 0.001) and OS (80.0% vs. 50.5%, *P* = 0.004) compared with IMRT alone. However, in patients with T3N0-1 disease, no significant survival differences were observed between patients treated with IMRT alone and CCRT (*P* > 0.05).

**Conclusions:**

CCRT is an effective therapy in stage III NPC, especially for patients with N2 disease, but IMRT alone may be adequate for N0-1 disease. Individualized treatment strategies are essential for patients with varying disease risks.

**Supplementary Information:**

The online version contains supplementary material available at 10.1186/s13014-021-01929-9.

## Introduction

Nasopharyngeal carcinoma (NPC) is a highly chemoradiosensitive tumor derived from the nasopharyngeal epithelium [1]. Stage I NPC can be treated with intensity-modulated radiotherapy (IMRT) alone, yielding a 5-year overall survival (OS) rate of more than 90% [2], while no consensus of treatment strategy for patients with stage II-IVA NPC (based on the 8th TNM staging system [3]) has been established. Thus, the National Comprehensive Cancer Network (NCCN) guidelines recommend patients with stage II–IVA NPC participate in clinical trials with a combination of chemotherapy and radiotherapy to acquire better treatment [4]. However, heterogeneity of the stages results in different survival outcomes, leading to investigations of optimal treatment strategies in different subpopulations.

As higher dose to the target and lower dose to organs at risks (OARs) can be achieved with IMRT compared with two-dimensional conventional radiotherapy (2DCRT) [5], better treatment outcomes have been achieved, especially in stage III NPC [6]. Retrospective studies demonstrated that concurrent chemotherapy (CCT) did not provide survival benefit in stage II and stage T3N0M0 NPC but more toxicities in the IMRT era [7, 8]. Moreover, patients with N1 stage disease did not acquire therapeutic gain from additional CCT in these studies. Stage III NPC includes disease with T3N0-1 and T1-3N2 stage, and the survival benefit of CCT in heterogeneous disease needs further assessment. To address this issue, we analyzed the long-term survival outcomes in patients with stage III NPC treated with IMRT alone or CCRT among subgroups of T3N0-1 and T1-3N2 disease.

## Materials and methods

### Patients

Patients with newly-diagnosed nonkeratinizing stage III NPC (according to the 8th TNM staging system) between February 2001 and December 2008 at the Sun Yat-sen University Cancer Center were reviewed. Inclusion criteria were as follows: [1] Karnofsky performance score ≥ 80; [2] age between 18 and 75 years old; [3] no history of cancer within 5 years; [4] receipt of IMRT; [6] no receipt of neoadjuvant chemotherapy, adjuvant chemotherapy, or target therapy. All clinical records including magnetic resonance imaging (MRI) materials were reviewed. The study was approved by the Medical Ethics Committee of Sun Yat-sen University Cancer Center, and written informed consent was waived due to the retrospective nature. Key data from this study has been uploaded onto the Research Data Deposit public platform (http://www.researchdata.org.cn; approval number: RDDA2019001373).

### Radiotherapy

All patients were treated with IMRT as previously reported. Target volumes and OARs were contoured according to the International Commission on Radiation Units and Measurements Reports 50 and 62 as well as our institutional treatment protocol [9]. Gross tumor volumes (GTVs) included GTVp (the primary gross tumor and metastatic retropharyngeal lymph node) and GTVnd (metastatic cervical lymph node). The clinical target volumes (CTVs) were contoured as CTV1 (high-risk regions: GTVp plus a 5–10 mm margin and the whole nasopharynx) and CTV2 (low-risk regions: CTV1 plus a 5–10 mm margin together with the bilateral cervical selective lymph drainage areas). The prescribed doses were 68, 60–66, 60, and 54 Gy in 30 fractions for the planning target volume (PTV) derived from GTVp, GTVnd, CTV1, and CTV2, respectively.

### Chemotherapy

The CCT regimens included [1] cisplatin alone regimen: 80 mg/m^2^ every three weeks for two to three cycles, or 30 mg/m^2^ weekly for four to six cycles; [2] PF regimen: combination of cisplatin (80 mg/m^2^) with 5-fluorouracil (750 mg/m^2^) every three weeks for two cycles, or a combination of nedaplatin (80 mg/m^2^) with 5-fluorouracil (750 mg/m^2^) every three weeks for two cycles; and [3] taxol alone regimen: 40 mg/m^2^ weekly for four to six cycles. Toxicities were evaluated according to the Common Terminology Criteria for Adverse Events (version 3.0).

### Follow-up

Follow-up was measured from the beginning of treatment to the last examination or death. Patients were assessed for the first three months, then every three months for three years, and every 6–12 months thereafter. The endpoints were OS, disease-free survival (DFS), distant metastasis-free survival (DMFS), and locoregional-free survival (LRFS), which referred to the time from treatment to death for any cause; to locoregional failure, distant failure, or death for any cause; to distant failure; and to locoregional failure, respectively.

### Statistical methods

The Mann–Whitney U test was used for ordinal variables, and the chi-squared test was used for categorical variables. The Kaplan–Meier method and log-rank test were used for survival analysis. A Cox regression model was used to identify the independent prognostic factors. Variables were assessed in the univariate analysis, and potential risk factors with statistical significance level of 0.2 were selected for multivariate analysis. SPSS 22.0 software (SPSS Inc., Chicago, IL, USA) and Stata 15.1 (Stata Corporation, College Station, TX, USA) were used for all analyses. A *P* value of less than 0.05 was considered statistically significant.

## Results

### Baseline characteristics

A total of 272 patients meeting the inclusion criteria were included in the study (Table 1). Of the entire cohort, the median age was 43 years old (range 15–75 years old). The distribution of patients between the IMRT alone group and CCRT group was well balanced except for gender (*P* = 0.020). Of the 272 patients, 190 patients received additional CCT; 132/190 (69.5%) patients received cisplatin alone, 32/190 (16.8%) patients received PF, and 26/190 (13.7%) patients received taxol alone.

**Table 1:** Baseline characteristics of patients with stage III NPC (N = 272)

Variables	IMRT alone (N = 82)	CCRT (N = 190)	*P*
*Sex*			0.020
Male	72 (87.8%)	143 (75.3%)	
Female	10 (12.2%)	47 (24.7%)	
*Age (year)*			0.303
≤ 43	38 (46.3%)	101 (53.2%)	
> 43	44 (53.7%)	89 (46.8%)	
*Smoking*			0.070
Yes	43 (52.4%)	113 (59.5%)	
No	39 (47.6%)	77 (40.5%)	
*Alcohol*			0.368
Yes	19 (23.2%)	155 (81.6%)	
No	63 (76.8%)	35 (18.4%)	
*T classification*			0.146
T1-2	12 (26.8%)	36 (18.9%)	
T3	60 (73.2%)	154 (81.1%)	
*N classification*			0.970
N0-1	49 (59.8%)	114 (60.0%)	
N2	33 (40.2%)	76 (40.0%)	
*EBV DNA*			0.398
< 2000	25 (30.5%)	68 (35.8%)	
≥ 2000	57 (69.5%)	122 (64.2%)	

NPC, nasopharyngeal carcinoma; IMRT, intensity-modulated radiation therapy; CCRT, concurrent chemoradiotherapy; EBV, Epstein–Barr virus

### Prognostic factors

Univariate and multivariate analyses (Tables 2 and 3) revealed that the N classification was significantly associated with DFS (hazard ratio [HR] 1.722, 95% confidence interval [CI] 1.115–2.657, *P* = 0.014). CCT was significantly associated with DFS (HR 1.661, 95% CI 1.064–2.594, *P* = 0.026) and OS (HR 1.876, 95% CI 1.141–3.083, *P* = 0.013). In addition, age (HR 1.946, 95% CI 1.161–3.264, *P* = 0.012) and smoking (HR 0.578, 95% CI 0.336–0.996, *P* = 0.048) were independent prognostic factors for OS.

**Table 2:** Univariate analysis of prognostic factors for 272 patients with stage III NPC

Variables	LRFS		DMFS		DFS		OS	
	HR (95% CI)	*P*	HR (95% CI)	*P*	HR (95% CI)	*P*	HR (95% CI)	*P*
Sex (male vs. female)	0.978 (0.400–2.392)	0.961	0.922 (0.462–1.840)	0.818	0.874 (0.499–1.530)	0.637	0.779 (0.407–1.491)	0.451
Age (≤ 43 vs. > 43, year)	1.254 (0.612–2.570)	0.537	1.224 (0.706–2.122)	0.471	1.450 (0.936–2.247)	0.096	2.087 (1.252–3.480)	0.005
Smoking (yes vs. no)	0.662 (0.323–1.357)	0.260	0.618 (0.356–1.073)	0.087	0.617 (0.399–0.953)	0.030	0.472 (0.286–0.781)	0.003
Alcohol (yes vs. no)	0.749 (0.321–1.746)	0.503	0.748 (0.392–1.430)	0.380	0.707 (0.427–1.170)	0.177	0.594 (0.341–1.035)	0.066
T classification (T1-2 vs. T3)	1.486 (0.371–2.014)	0.735	1.580 (0.445–1.623)	0.622	1.268 (0.520–1.451)	0.590	1.069 (0.581–1.966)	0.830
N classification (N0-1 vs. N2)	1.890 (0.923–3.873)	0.082	1.672 (0.965–2.895)	0.067	1.670 (1.083–2.575)	0.020	1.506 (0.922–2.459)	0.102
CCT (yes vs. no)	1.721 (0.828–3.575)	0.146	1.485 (0.842–2.620)	0.172	1.721 (1.106–2.678)	0.016	1.976 (1.205–3.239)	0.007
EBV DNA (< 2000 vs. ≥ 2000)	1.747 (0.750–4.071)	0.196	1.398 (0.756–2.585)	0.286	1.084 (0.684–1.718)	0.732	1.081 (0.649–1.802)	0.764

NPC, nasopharyngeal carcinoma; HR, hazard ratio; CI, confidence interval; LRFS, locoregional-free survival; DMFS, distant metastasis-free survival; DFS, disease-free survival; OS, overall survival; CCT, concurrent chemotherapy; EBV, Epstein–Barr virus

**Table 3:** Multivariate analysis of prognostic factors for 272 patients with stage III NPC

Variables	LRFS		DMFS		DFS		OS	
	HR (95% CI)	*P*	HR (95% CI)	*P*	HR (95% CI)	*P*	HR (95% CI)	*P*
Age (≤ 43 vs. > 43, year)	–	–	–	–	1.378 (0.884–2.148)	0.157	1.946 (1.161–3.264)	0.012
Smoking (yes vs. no)	–	–	–	–	0.728 (0.452–1.172)	0.192	0.578 (0.336–0.996)	0.048
Alcohol (yes vs. no)	–	–	0.643 (0.369–1.121)	0.120	0.872 (0.506–1.502)	0.621	0.839 (0.462–1.522)	0.563
N classification (N0-1 vs. N2)	1.910 (0.930-3.920)	0.078	1.683 (0.972–2.916)	0.063	1.722 (1.115–2.657)	0.014	1.629 (0.996–2.665)	0.052
CCT (yes vs. no)	1.737 (0.834-3.617)	0.140	1.431 (0.808–2.535)	0.219	1.661 (1.064–2.594)	0.026	1.876 (1.141–3.083)	0.013
EBV DNA (< 2000 vs. ≥ 2000)	1.664 (0.713-3.883)	0.239	–	–	–	–	–	–

NPC, nasopharyngeal carcinoma; HR, hazard ratio; CI, confidence interval; LRFS, locoregional-free survival; DMFS, distant metastasis-free survival; DFS, disease-free survival; OS, overall survival; CCT, concurrent chemotherapy; EBV, Epstein–Barr virus

### Survival outcome

**Fig. 1:**
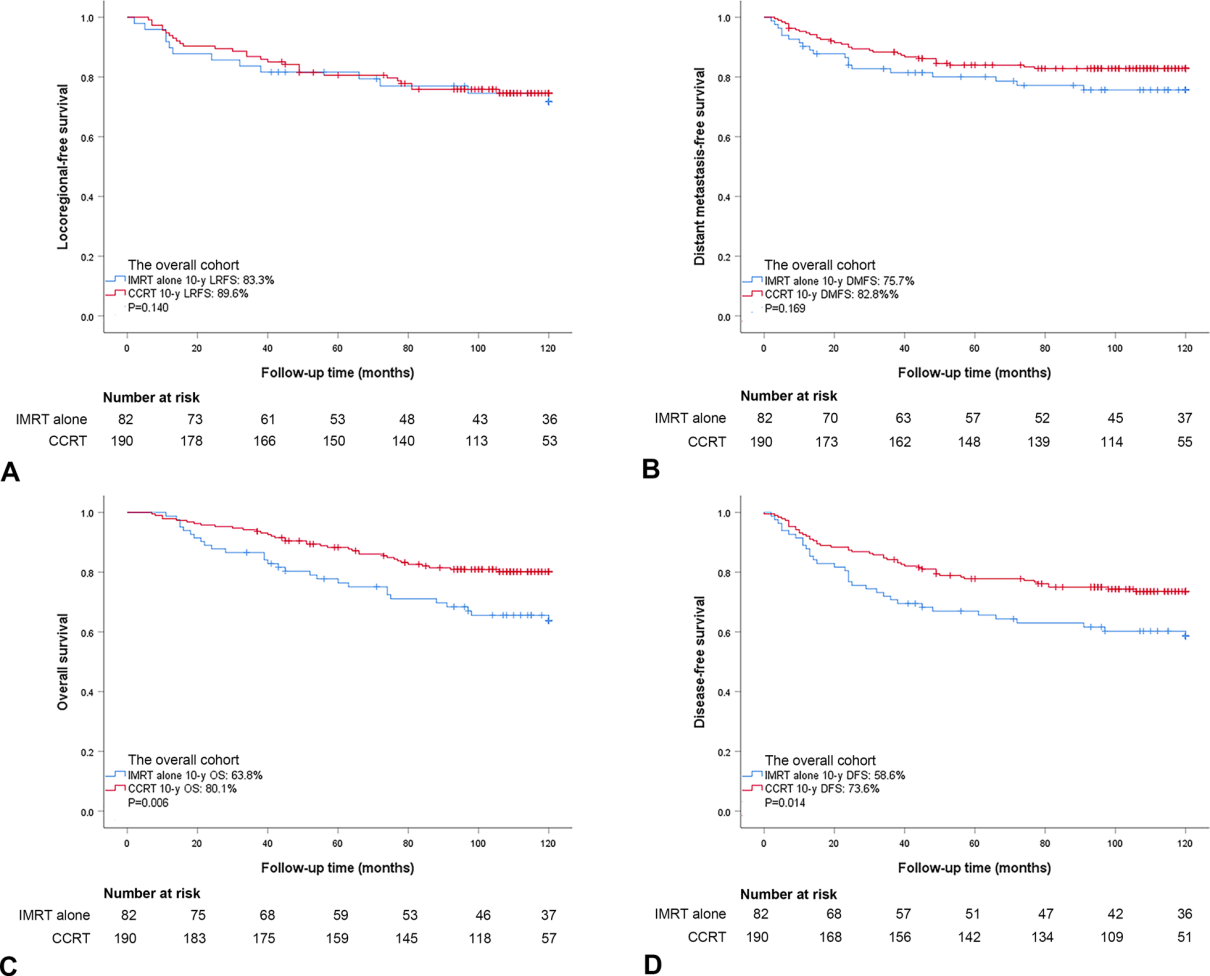
Kaplan–Meier estimates of locoregional-free survival (**A**), distant metastasis-free survival (**B**), overall survival (**C**), and disease-free survival (**D**) for the entire cohort treated with IMRT alone and CCRT. *P*-values were calculated by log-rank test. IMRT, intensity-modulated radiotherapy; CCRT, concurrent chemoradiotherapy; LRFS, locoregional-free survival; DMFS, distant metastasis-free survival; OS, overall survival; and DFS, disease-free survival

The median follow-up time was 108 months (range 7–180 months). By the last follow-up, 72 patients had treatment failures, including 20 with locoregional relapse alone (15 with local relapse, 3 with regional relapse, 2 with locoregional relapse), 42 with distant metastasis alone, and 10 with both locoregional relapse and distant metastasis. Moreover, 67 patients had died at the time of the last follow-up. Specifically, 44 died of distant metastasis alone, 7 died of locoregional relapse alone, 3 died of nasopharyngeal hemorrhage, and 13 died of other reasons (i.e., accidents). Of the entire cohort, the 10-year LRFS, DMFS, DFS, and OS were 87.8%, 80.7%, 68.8%, and 74.9%, respectively. Significant differences were observed between individuals treated with IMRT alone and CCRT in 10-year OS (63.8% vs. 80.1%, *P* = 0.006) and DFS (58.6% vs. 73.6%, *P* = 0.014), while no significant differences were observed in 10-year LRFS (83.3% vs. 89.6%, *P* = 0.140) and DMFS (75.7% vs. 82.8%, *P* = 0.169, Fig. 1).

**Fig. 2:**
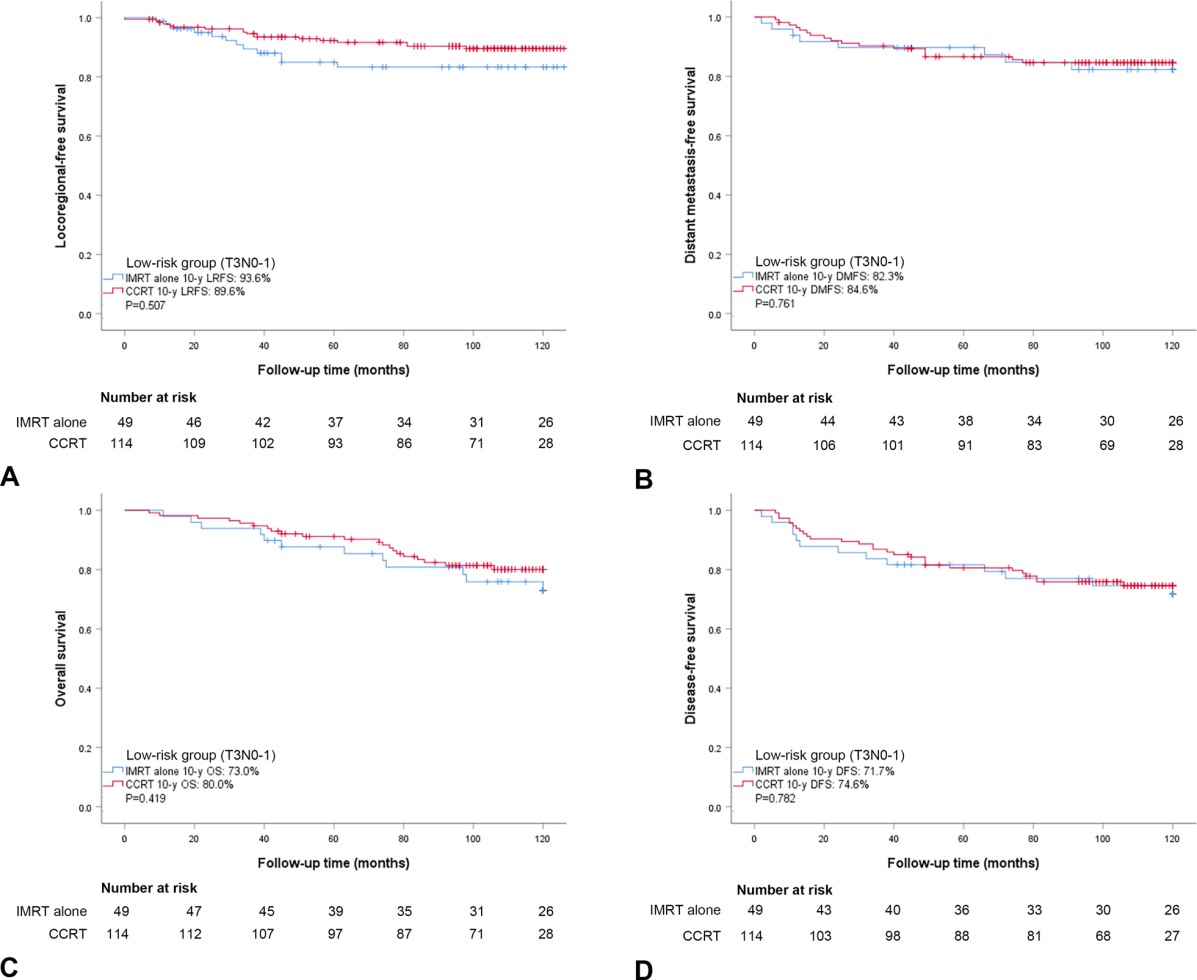
Kaplan–Meier estimates of locoregional-free survival (**A**), distant metastasis-free survival (**B**), overall survival (**C**), and disease-free survival (**D**) for the low-risk group (T3N0-1) treated with IMRT alone and CCRT. *P*-values were calculated by log-rank test. IMRT, intensity-modulated radiotherapy; CCRT, concurrent chemoradiotherapy; LRFS, locoregional-free survival; DMFS, distant metastasis-free survival; OS, overall survival; and DFS, disease-free survival

**Fig. 3:**
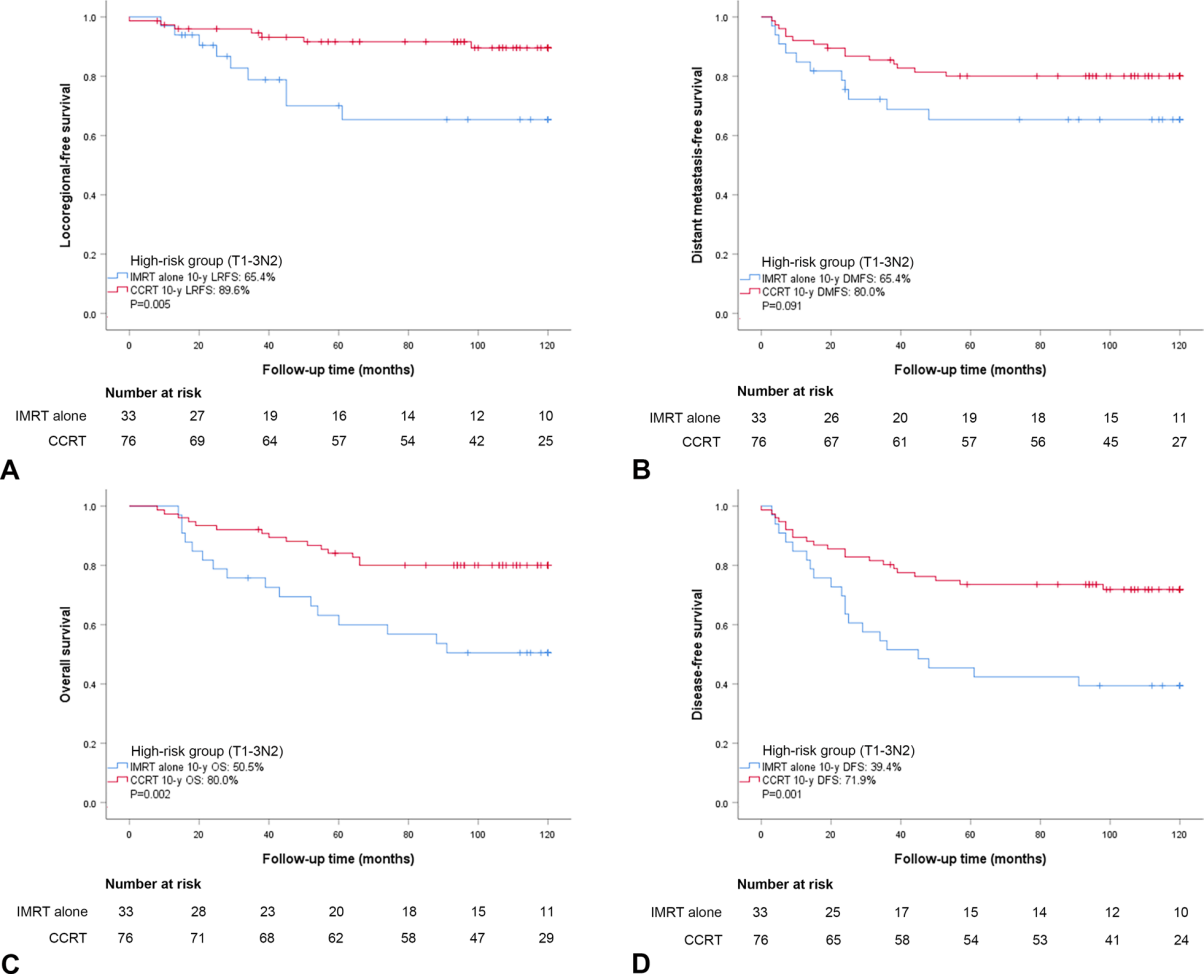
Kaplan–Meier estimates of locoregional-free survival (**A**), distant metastasis-free survival (**B**), overall survival (**C**), and disease-free survival (**D**) for the high-risk group (T1-3N2) treated with IMRT alone and CCRT. *P*-values were calculated by log-rank test. IMRT, intensity-modulated radiotherapy; CCRT, concurrent chemoradiotherapy; LRFS, locoregional-free survival; DMFS, distant metastasis-free survival; OS, overall survival; and DFS, disease-free survival

We also evaluated survival outcomes in the low-risk group (stage T3N0-1, n = 163) and the high-risk group (stage T1-3N2, n = 109) when treated with IMRT alone or CCRT. In the low-risk group, the baseline characteristics were well balanced between the IMRT alone group and CCRT group except for sex (*P* = 0.037) (Additional file 1: Table S1). No significant survival differences were observed between patients who received IMRT alone (n = 49) and CCRT (n = 114), including LRFS, DMFS, DFS, and OS (*P* > 0.05, Fig. 2). In the high-risk group, the baseline characteristics were well balanced between the IMRT alone group and CCRT group except for alcohol consumption (*P* = 0.040) (Additional file 1: Table S1). Significant survival differences were observed between patients who received IMRT alone (n = 33) and CCRT (n = 76), including LRFS (65.4% vs. 89.6%, *P* = 0.005), DFS (39.4% vs. 71.9%, *P* = 0.001), and OS (50.5% vs. 80.0%, *P* = 0.002). Interestingly, no significant difference was observed in DMFS (65.4% vs. 80.0%, *P* = 0.091) between patients who received IMRT alone and CCRT in the high-risk group (Fig. 3).

### Toxicity

The incidences of Grade 3–4 acute adverse events (AEs) in 272 patients are listed in Table 4. Patients treated with CCRT experienced more hematological AEs, including leukopenia (11.6% vs. 1.2%, *P* = 0.005), neutropenia (7.4% vs. 0.0%, *P* = 0.012), and thrombocytopenia (4.8% vs. 0.0%, *P* = 0.045), than those treated with IMRT alone except for anemia (2.1% vs. 0.0%, *P* = 0.186). Moreover, a higher incidence of mucositis was observed in the CCRT group compared with the IMRT alone group (50.5% vs. 19.5%, *P *< 0.001), while no statistical differences were observed in xerostomia, dermatitis, nausea/vomiting, and hepatoxicity between the two groups (*P* > 0.05).

**Table 4:** Grade 3–4 acute adverse events in 272 patients with stage III NPC

Variables	IMRT (N = 82)	CCRT (N = 190)	*P*
*Hematological*			
Leukopenia	1 (1.2%)	22 (11.6%)	0.005
Neutropenia	0 (0.0%)	14 (7.4%)	0.012
Anemia	0 (0.0%)	4 (2.1%)	0.186
Thrombocytopenia	0 (0.0%)	9 (4.8%)	0.045
*Non-hematological*			
Mucositis	16 (19.5%)	96 (50.5%)	< 0.001
Xerostomia	0 (0.0%)	4 (2.1%)	0.186
Dermatitis	3 (3.7)	6 (3.2%)	0.833
Nausea/vomiting	0 (0.0%)	5 (2.6%)	0.139
Hepatoxicity	1 (1.2%)	0 (0.0%)	0.128

NPC, nasopharyngeal carcinoma; IMRT, intensity-modulated radiation therapy; CCRT, concurrent chemoradiotherapy

## Discussion

A previous meta-analysis showed that CCT brought survival benefit to patients with locoregionally advanced NPC in the context of 2DCRT, even in patients with stage II disease [10]. Thus, radiotherapy combined with chemotherapy has been guideline-recommended in NPC except for stage I disease [4], since additional CCT is regarded as the main contributor to improved radiosensitivity. However, the development of IMRT, which allows sharper radiation dose gradients between tumor and critical organs, provides improved locoregional control and reduced toxicities in the treatment of NPC [6]. Therefore, it is necessary to re-assess the relative gain of additional CCT in heterogeneous disease treated with IMRT.

Investigators have reported the efficacy of CCRT in NPC in the IMRT era with controversial results. Sun et al. [11] and Yi et al. [12] did not observe survival improvement in locoregionally advanced NPC when treated with CCRT, while researchers from Hong Kong observed enhanced treatment outcomes in NPC with additional CCT of cisplatin [13]. This inconsistency might result from the heterogeneity of enrolled patients, who were staged at II–IV. Pan and colleagues [14] found similar survival outcomes between the IMRT alone group and CCRT group (*P* > 0.05) in stage II NPC, indicating that the benefit of additional CCT might contribute little to the already excellent locoregional control. Moreover, Zhang et al. [7] failed to identify CCT as an independent prognostic factor in stage T3N0M0 and stage II NPC, and subgroup analysis revealed that CCT did not provide a significant survival difference in patients with N1 disease (*P* > 0.05). Similar results were observed by Aftab and colleagues [8]. Thus, although the combination of radiotherapy and chemotherapy is recommended in stage III NPC, the role of CCT will require further assessment.

To the best of our knowledge, this is the first comparison study evaluating IMRT alone and CCRT in stage III NPC. In the multivariate analysis, N classification was the main independent prognostic factor for DFS (HR 1.722, 95% CI 1.115–2.657, *P* = 0.014). Of the entire cohort, 10-year OS (63.8% vs. 80.1%; *P* = 0.006) and DFS (58.6% vs. 73.6%; *P* = 0.014) were better in the CCRT group compared with the IMRT alone group. This indicated that patients with stage III disease could benefit from additional CCT, which is consistent with previous studies of locoregionally advanced cases [15, 16]. However, when we divided patients into a low-risk group of staged T3N0-1disease and high-risk group of staged T1-3N2 disease to address this issue, the survival differences were not statistically significant when patients were treated with IMRT alone and CCRT, including OS, DFS, DMFS, and LRFS (*P* > 0.05), in the low-risk subgroup. By contrast, in the high-risk group, the 10-year LRFS (65.4% vs. 89.6%, *P* = 0.005), DFS (39.4% vs. 71.9%, *P* = 0.001), and OS (50.5% vs. 80.0%, *P* = 0.002) were significantly better when patients were treated with CCRT rather than IMRT alone except for DMFS (65.4% vs. 80.0%, *P* = 0.091).

There are several potential explanations for our findings. Firstly, the benefit of locoregional control from additional CCT might need to be re-assessed in the IMRT era. In the present study, comparable LRFS was observed in patients treated with IMRT alone and CCRT (93.6% vs. 89.6%, *P* = 0.507) in the low-risk group. We propose that the substantial benefit of CCT might be narrowed with application of IMRT in disease with low tumor burden. By contrast, worse LRFS was observed in patients treated with IMRT alone than CCRT (65.4% vs. 89.6%, *P* = 0.005) in the high-risk group, indicating that additional CCT might still be effective in patients with high tumor burden because of its ability to improve radiosensitivity.

Secondly, the present study failed to identify significant improvement in DMFS in the whole cohort comparing CCRT with IMRT alone (82.8% vs. 75.7%, *P* = 0.169). Although there was a trend of better DMFS in the high-risk group when treated with CCRT (IMRT alone vs. CCRT: 65.4% vs. 80.0%, *P* = 0.091), statistical significance was not observed. Further explorations of combination therapy for patients in the high-risk group are expected to reduce distant metastasis.

Thirdly, individualized treatment strategies need to be planned based on stratified risk of locoregional failure and distant failure. In clinical practice, stage III-IVA NPC disease is regarded as locoregional advanced NPC, and similar treatments are recommended [4]. However, previous studies demonstrated that prognosis of patients with stage III NPC was better than that of patients with stage IVA NPC [3]. Moreover, investigators reported no significant survival difference between the stage T3N0M0 subgroup and stage II subgroup, and IMRT alone was effective [7]. Thus, the treatment failure risk in stage III disease varies. Consistent with previous results [17], the present study revealed that stage III NPC treated with IMRT had different risk of treatment failures depending on the N stage. Therefore, if similar treatment strategies are provided for stage III disease, intensive treatment might be an aggressive approach in the low-risk group, while patients in the high-risk group might receive insufficient treatment.

On the other hand, increased Grade 3–4 hematological AEs were observed in the CCRT group compared with the IMRT alone group (*P* < 0.05) except for anemia (*P* = 0.186). Moreover, the incidence rate of Grade 3–4 mucositis was higher in the CCRT group compared with the IMRT alone group (*P* < 0.001). We postulated that the differences might result from the additional CCT in the CCRT group. However, no significant differences were observed between the two treatment groups regarding other non-hematological Grade 3–4 AEs such as xerostomia and dermatitis. We attributed the similar incidence of radiotherapy-related AEs in the two groups to the advanced IMRT techniques. Therefore, additional CCT might not provide significant survival benefit but increased Grade 3–4 hematological toxicities in the low-risk group, and the routine use of CCT in this subgroup needs further evaluation.

There are several limitations of this study. This is a retrospective study with a small sample size. The IMRT technique was introduced in our institution in 2001. Therefore, the number of patients with stage III disease who received IMRT between 2001 and 2008 is limited. The efficacy of CCT in locoregionally advanced NPC achieved a consensus in 2006 based on two meta-analyses [10, 18]. Therefore, the treatment strategy for patients with stage III disease was not uniform before 2008, and rare patients with stage III disease received IMRT alone after 2008 in our institution. However, the data during those years is valuable, and allowed us to conduct the current retrospective study assessing the efficacy of CCT in subgroups of stage III disease. Moreover, the dataset in the present study was from the Asian population, in which NPC is prevalent with a high rate of Epstein-Barr virus (EBV) infection. The validity of the current findings in non-endemic, non-EBV related regions needs further exploration. Of note, this study was based on long-term follow-up of real-world data, with a focus on a special subpopulation of NPC. Therefore, our results provide a basis for future research of individualized treatment modalities for stage III NPC.

## Conclusions

Our results demonstrated that CCRT was able to improve survival outcomes in stage III NPC, especially for patients with N2 disease, while survival benefit of additional CCT in low-risk subgroup with N0-1 disease was limited with increased toxicities. Further randomized trials are warranted to confirm the results of our study.

## Supplementary Information

**Additional file 1**. **Table S1**. Baseline characteristics of patients with stage III NPC in N0-1 and N2 subgroups.

## Data Availability

All data generated or analyzed during this study are included in this published article.

## Abbreviations

NPC: Nasopharyngeal carcinoma
IMRT: Intensity-modulated therapy
2DCRT: Two-dimensional conventional radiotherapy
CCRT: Concurrent chemoradiotherapy
CCT: Concurrent chemotherapy
HR: Hazard ratio
CI: Confidence interval
NCCN: National Comprehensive Cancer Network
MRI: Magnetic resonance imaging
OS: Overall survival
LRFS: Locoregional recurrence-free survival
DMFS: Distant metastasis-free survival
DFS: Disease-free survival
OARs: Organs at risk
GTV: Gross tumor volume
GTVp: Primary gross tumor (including retropharyngeal lymph node metastases)
GTVnd: Cervical lymph node metastasis
CTV1: High-risk clinical target volume
CTV2: Low-risk clinical target volume
PTV: Planning target volume
PF: Combination of cisplatin (80 mg/m^2^) with 5-fluorouracil (750 mg/m^2^), or combination of nedaplatin (80 mg/m^2^) with 5-fluorouracil (750 mg/m^2^)
AE: Acute adverse event
