# Supplementary material for: Efficacy of concurrent chemoradiotherapy in subgroups of stage III nasopharyngeal carcinoma: an analysis based on 10-year follow-up
Source: Radiat Oncol. 2021 Nov 6;16:215. doi: 10.1186/s13014-021-01929-9 (PMC8571837; doi:10.1186/s13014-021-01929-9)
Supplement: Supplementary file 1 — Additional file 1. Table S1. Baseline characteristics of patients with stage III NPC in N0-1 and N2 subgroups. [file 13014_2021_1929_MOESM1_ESM.docx]

**Additional file 1**

**Table S1 Baseline characteristics of patients with stage III NPC in N0-1 and N2 subgroups**

| **Variables** | **N0-1 (N=163)** | | | **N2 (N=109)** | | |
| --- | --- | --- | --- | --- | --- | --- |
|  | **IMRT alone (N=49)** | **CCRT (N=114)** | **P** | **IMRT alone (N=33)** | **CCRT (N=76)** | **P** |
| Sex |  |  | 0.037 |  |  | 0.256 |
| Male | 44 (89.8) | 86 (75.4) |  | 28 (84.8) | 57 (75.0) |  |
| Female | 5 (10.2) | 28 (24.6) |  | 5 (15.2) | 19 (25.0) |  |
| Age (year) |  |  | 0.987 |  |  | 0.108 |
| ≤43 | 24 (49.0) | 56 (49.1) |  | 14 (42.4) | 45 (59.2) |  |
| >43 | 25 (51.0) | 58 (50.9) |  | 19 (57.6) | 31 (40.8) |  |
| Smoking |  |  | 0.135 |  |  | 0.302 |
| Yes | 26 (53.1) | 46 (40.4) |  | 17 (51.5) | 31 (40.8) |  |
| No | 23 (46.9) | 68 (59.6) |  | 16 (48.5) | 45 (59.2) |  |
| Alcohol |  |  | 0.567 |  |  | 0.040 |
| Yes | 8 (16.3) | 23 (20.2) |  | 11 (33.3) | 12 (15.8) |  |
| No | 41 (83.7) | 91 (79.8) |  | 22 (66.7) | 64 (84.2) |  |
| T classification |  |  | 1.000 |  |  | 0.065 |
| T1-2 | - | - |  | 22 (66.7) | 36 (47.4) |  |
| T3 | 49 (100.0) | 114 (100.0) |  | 11 (33.3) | 40 (52.6) |  |
| N classification |  |  | 1.000 |  |  | 1.000 |
| N0-1 | 49 (100.0) | 114 (100.0) |  | - | - |  |
| N2 | - | - |  | 33 (100.0) | 76 (100.0) |  |
| EBV DNA |  |  | 0.757 |  |  | 0.223 |
| <2000 | 17 (34.7) | 43 (37.7) |  | 8 (24.2) | 25 (32.9) |  |
| ≥2000 | 32 (65.3) | 71 (62.3) |  | 25 (75.8) | 51 (67.1) |  |

**Abbreviations:** NPC, nasopharyngeal carcinoma; IMRT, intensity-modulated radiation therapy; CCRT, concurrent chemoradiotherapy; EBV, Epstein–Barr virus.
